# Evaluation of *Rint1* as a modifier of intestinal tumorigenesis and cancer risk

**DOI:** 10.1371/journal.pone.0172247

**Published:** 2017-03-06

**Authors:** Karla L. Otterpohl, Karen A. Gould

**Affiliations:** Department of Genetics, Cell Biology and Anatomy, University of Nebraska Medical Center, Omaha, Nebraska, United States of America; CNR, ITALY

## Abstract

The Rad50 Interacting Protein 1 (*Rint1*) influences cellular homeostasis through maintenance of endoplasmic reticulum, Golgi and centrosome integrity and regulation of vesicle transport, autophagy and the G_2_/M checkpoint. Rint1 has been postulated to function as a tumor suppressor as well as an oncogene, with its role depending perhaps upon the precise cellular and/or experimental context. In humans, heterozygosity for germline missense variants in *RINT1* have, in some studies, been associated with increased risk of both breast and Lynch syndrome type cancers. However, it is not known if these germline variants represent loss of function alleles or gain of function alleles. Based upon these findings, as well as our initial consideration of *Rint1* as a potential candidate for *Mom5*, a genetic modifier of intestinal tumorigenesis in *Apc*^*Min/+*^ mice, we sought to explicitly examine the impact of *Rint1* on tumorigenesis in *Apc*^*Min/+*^ mice. However, heterozygosity for a knockout of *Rint1* had no impact on tumorigenesis in *Rint1*^*+/-*^; *Apc*^*Min*/+^ mice. Likewise, we found no evidence to suggest that the remaining *Rint1* allele was lost somatically in intestinal tumors in *Apc*^*Min*/+^ mice. Interestingly, in contrast to what has been observed in *Rint1*^*+/-*^ mice on a mixed genetic background, *Rint1*^*+/-*^ mice on a pure C57BL/6J background did not show spontaneous tumor development. We also evaluated colorectal cancer data available in the COSMIC and ONCOMINE databases and found that *RINT1* overexpression, as well as the presence of somatic missense mutations in *RINT1* were associated with colorectal cancer development. *In vitro* evaluation of two missense variants in *RINT1* suggested that such variants do have the potential to impact RINT1 function.

## Introduction

The Rad50 interacting protein 1 (Rint1), was identified through its interaction with Rad50, and found to be involved in control of the G_2_/M checkpoint in response to DNA damage [1]. Depletion of Rint1 *in vitro* results in genomic instability, failure to complete the G2/M checkpoint and ultimately cell death [2, 3]. Rint1 has also been shown to function in a variety of other cellular processes, including telomerase-independent telomere elongation, centrosome duplication, endoplasmic reticulum (ER) and Golgi homeostasis, ER-Golgi vesicle trafficking, ER stress and autophagy[3–8]. Homozygosity for a *Rint1* knockout allele in mice results in embryonic lethality during early postimplantation development, apparently due to a failure of the blastocysts to proliferate [2]. However, selective deletion of *Rint1* in non-dividing Purkinje cells also results in cell death [3]. However, in this later case, cell death appeared to be the result of defects in ER-Golgi function and inhibition of autophagy [3].

Several studies have provided evidence to suggest that heterozygosity for mutations in the *Rint1* gene is associated with tumorigenesis. Approximately 80% of mice heterozygous for the *Rint1* knockout allele (*Rint1* ^*tm1Whl/+*^) were reported to develop spontaneous lymphomas or solid tumors affecting a variety of tissues including the liver, lung, and uterus [2]. However, the average life span of these mice was 2 years, indicating that tumors developed with a long latency period. This study suggests that *Rint1* may function as a general tumor suppressor gene. By contrast, another study identified *Rint1* as a potential oncogene in glioblastoma [9]. Over-expression of *Rint1* promoted anchorage-independent growth of glioblastoma cell lines, whereas shRNA knockdown of *Rint1* significantly reduced cell viability. Most recently, germline variants in *Rint1* have been associated with increased risk for breast cancer as well as Lynch syndrome type cancers, including colorectal, endometrial, and gastric cancers [10]. Whole exome sequencing in families with previously unexplained hereditary breast cancer identified three different germline *Rint1* variants, each found in a single family [10]. Focused analysis subsequently identified more than 20 different rare germline variants in *RINT1* predicted to be damaging that were associated with breast cancer in a case control study and 5 additional predicted damaging germline *RINT1* variants associated with breast, colorectal, endometrial and gastric cancer in six cancer–prone families [10]. Interestingly, the vast majority of the predicted damaging germline variants in *RINT1* that were associated with increased risk of breast and Lynch syndrome type cancers in this study were missense variants. The potential impact of these variants on Rint1 function is unknown. However, it should be noted that a recent study found no evidence for an association between germline missense variants in *RINT1* and either breast or Lynch syndrome type cancer risk [11].

Our laboratory initially identified *Rint1* as a potential candidate for the *Mom5* modifier of intestinal neoplasia in *Apc*^*Min/+*^ mice [12]. The B6 allele of the *Mom5* locus is associated with increased intestinal adenoma number and size, and the B6 allele of *Rint1* harbors two missense mutations that are similar to some of the *RINT1* variants linked to breast and Lynch syndrome type cancers in humans. Both variants in the B6 allele result in the substitution of a highly conserved isoleucine residue in the coiled-coil domain of Rint1 [12]and thus have the potential to alter the folding of the coiled-coil of Rint1, the ability of Rint1 to interact with its binding partners and/or alter the stability of the Rint1 protein. Rint1 interacts with several proteins via its coiled-coil domain, including Zw10, Uvrag, Cog1, and Stx16 [6, 7, 13]. All these proteins are involved in membrane trafficking between the ER and Golgi. Although fine structure mapping ultimately excluded *Rint1* as a candidate for *Mom5* [14], because *RINT1* mutations had been associated with increased risk of Lynch syndrome type cancers in humans, we were interested in explicitly examining the impact of *Rint1* mutation on intestinal tumorigenesis in *Apc*^*Min*/+^ mice. Although these *in vivo* studies suggested that heterozygosity for a knockout of *Rint1* did not enhance intestinal tumorigenesis in *Apc*^*Min*/+^ mice, *in vitro* studies indicated that missense mutations in *Rint1* do have the potential to impact protein-protein interactions involving *Rint1*. We also found that over-expression of *RINT1* and somatic missense mutations in *RINT1* were relatively frequent in human colorectal cancers whereas loss of *RINT1* or *RINT*1 expression were infrequent events in these cancers.

## Materials and methods

### Care and production of B6 mice congenic for the *Rint1* knockout allele

All mice used were housed in facilities at the University of Nebraska Medical Center accredited by the American Association for Accreditation of Laboratory Animal Care. Mice were kept in a climate controlled environment with 14-hour light/10-hour dark cycles and access to Harlan irradiated rodent diet 7904 (Harlan Teklad, Madison, WI) and water *ad libitum*. All procedures involving live mice were approved by the University of Nebraska Medical Center Institutional Animal Care and Use Committee.

**Fig 1:**
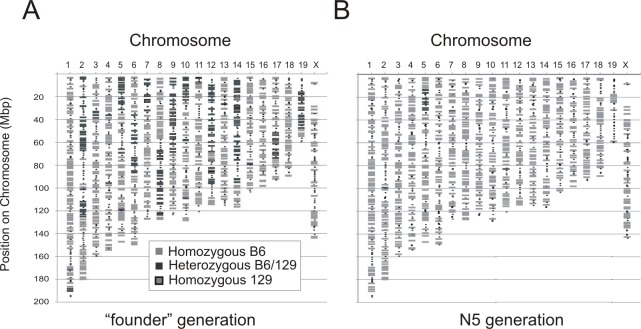
Evaluation of the genetic background of *Rint1*^*+/-*^ mice. Genome-wide SNP genotyping of the four founder *Rint1*^*tm1WHL*^ mice, each of which was heterozygous for the *Rint1* knockout allele, was performed. A representative example is shown (A) revealing a highly mixed 129 x B6 genetic background. After marker assisted selection using simple sequence repeat markers, genome-wide SNP genotyping was again performed on a group of *Rint1*^*+/-*^ mice (N = 4). A representative example is shown (B) clearly demonstrating that 129 alleles unlinked to the *Rint1* knockout allele have been eliminated.

Four *Rint1*^*tm1Whl/+*^ (*Rint1*^*+/-*^) mice, heterozygous for a targeted disruption of exons 5 and 6 of *Rint1* (Lin et al. 2007), were obtained from Dr. Wen Hwa Lee (University of California-Irvine, Irvine, California). These mice were on a mixed 129SvEv (129) and C57BL/6J (B6) genetic background. Thus, the genotype at SNPs distributed throughout the genome was determined at the DartMouse™ Speed Congenic Core Facility at the Geisel School of Medicine at Dartmouth University using the Illumina, Inc. (San Diego, CA) GoldenGate Genotyping Assay. The raw SNP data were analyzed using SNaP-Map™ and Map-Synth™ software. Analysis of the genetic background of the founder mice revealed that all mice carried residual 129 alleles spanning a 32 Mbp region linked to the *Rint1* knockout allele (Fig 1A). Additionally, these mice carried residual 129 alleles unlinked to *Rint1* at markers on 13 other autosomes (Fig 1A). Therefore, the *Rint1* knockout allele was transferred to a uniform B6 genetic background through serial backcrossing in conjunction with marker assisted selection with polymorphic simple sequence repeat markers spanning the regions of residual heterozygosity (Table 1). Mice from the fifth backcross generation were again evaluated by the Dartmouse Speed Congenic Facility and were found to be free of residual 129 alleles unlinked to the *Rint1* knockout allele (Fig 1B). B6.*Rint1*^*+/-*^ females were then crossed to B6.*Apc*^*Min/+*^ males for determination of the impact of heterozygosity for the *Rint1* knockout allele on intestinal tumorigenesis on a uniform B6 background. Mice that were heterozygous for the *Rint1* knockout allele and the A*pc*^*Min*^ mutation were identified by previously described PCR based genotyping assays [2, 15].

**Table 1:** Marker used for marker assisted backcrossing of B6.129Sv-*Rint1tm1WHL* mice ^1^.

Genetic Marker	Location (cM; Mbp)	Genetic Marker	Location (cM; Mbp)
*D1mit17*	95.0; 189.5	*D6Mit261*	39.5; 88.5
*D2mit312*	1.8; 3.2	*D6Mit373*	77.7; 147.0
*D2Mit156*	31.6; 57.0	*D8Mit45*	42.1; 87.3
*D2Mit13*	55.6; 106.4	*D8Mit166*	54.8; 110.9
*D3Mit21*	18.3; 37.1	*D9Mit250*	2.4; 8.3
*D5Mit346*	2.6; 4.8	*D9Mit21*	31.3; 57.6
*D5Mit294*	9.8; 21.3	*D10Mit152*	3.7; 12.1
*D5Mit348*	11.9; 24.9	*D11Mit71*	4.7; 6.9
*D5Mit176*	15.8; 30.0	*D12MIt182*	5.5; 10.8
*D6Mit77*	15.27; 35.3	*D12Mit12*	8.4; 24.6
*D6Mit33*	24.4; 50.8	*D12Mit60*	15.5; 34.7
*D6Mit316*	27.4; 55.5	*D14Mit52*	19.4; 32.4
*D6Mit123*	27.7; 57.6	*D14Mit60*	24.6; 47.0
*D6Mit94*	29.5; 62.6	*D14Mit39*	35.6; 68.5
*D6Mit188*	32.5; 75.4	*D17Mit180*	26.7; 51.4
*D6Mit3*	34.8; 78.6	*D19Mit119*	34.0; 40.2
*D6Mit9*	37.9; 87.4		

^1^ Only markers contained within regions of heterozygosity identified in founder B6.129Sv-*Rint1*^*tm1WHL*^ mice were used for marker assisted selection. Markers in regions already homozygous for the B6 allele were not evaluated.

### Evaluation of spontaneous tumorigenesis in *Rint1*^*+/-*^ mice

*Rint1*^*+/-*^ and *Rint1*^*+/+*^ mice were sacrificed at 450 days of age and all organs were examined for evidence of macroscopic tumor development. Solid organs were then collected and fixed in 10% neutral buffered formalin (Fischer Scientific, Kalamazoo, MI) to assess the presence of microscopic tumors. Fixed tissue was processed, embedded, sectioned and stained with Hemotoxylin and Eosin. Sections were analyzed by light microscopy.

### Evaluation of embryonic lethality in *Rint1*^*-/-*^ mice

*Rint1*^*+/-*^ mice were intercrossed and the resulting offspring were genotyped at *Rint1*. The observed genotype frequencies were compared to those expected by chi square analysis using SPSS software (version 21, IBM, New York, NY).

### Evaluation of intestinal tumor number and size

The intestinal tract was collected from 90 day old *Apc*^*Min/+*^ mice. The small intestine was divided into four equal parts, slit open, cleaned with phosphate-buffered saline solution (PBS), and fixed in 10% neutral buffered formalin. The colon was also cleaned and fixed in formalin. After 48 hours, the formalin was exchanged for 70% ethanol. Tumors from each segment of the small intestine and the large intestine were counted under a Leica MZ16FA dissecting microscope (Leica Microsystems, Heerbrugg, Switzerland). To determine tumor size, up to 20 tumors from each segment of the small intestine were measured using an eye-piece reticle and the Leica dissecting microscope. Genotype-phenotype correlation was determined using the Mann-Whiney U test in SPSS.

### Analysis of *Rint1* expression in intestinal tumors

Pedunculate colon tumors from *Apc*^*Min/+*^ mice were snipped from the underlying mucosa and frozen in liquid nitrogen. Adjacent normal mucosa, collected by scraping with a glass slide, was also snap frozen in liquid nitrogen. RNA from tumors and normal colonic mucosa was isolated using the Absolutely RNA Miniprep Kit from Agilent Technologies (Santa Clara, CA). RNA was used as a template to make cDNA using the SuperScript VILO Master Mix kit (Life Technologies, Grand Island, NY). The cDNA was then used in a quantitative RT-PCR assay with Sybr Green Master Mix (Life Technologies, Grand Island, NY) to examine the expression of the *Rint1* transcript. RNA from B6 small intestine epithelial cells was used as a control to generate a standard curve to quantitate the amplified products. Relative expression was determined by normalizing values to *Gapdh*. Primers used include Rint1 forward: 5’-TTTTCCCGTGGCCTTGTGAT-3’, Rint1 reverse: 5’-AAACAGCAGCACCTCATCCA-3’, Gapdh forward: 5’-TGCACCACCAACTGCTTAG-3’, and Gapdh reverse: 5’GGATGCAGGGATGATGTT-3’. Statistical significance for qRT-PCR was determined using the paired samples t-test in SPSS.

### Bioinformatic analysis of *RINT1* expression and somatic mutation in human colorectal cancers

*RINT1* transcript expression in normal colorectal tissues and colorectal cancers were compared using The Cancer Genome Atlas dataset in the ONOCOMINE database (www.oncomine.org). The threshold used to obtain the most significant *RINT1* probes for analysis was set at a two-fold difference in expression between cancers and normal tissues with a *P*-value < 1 × 10−4. The Catalog of Somatic Mutations in Cancer (COSMIC) database (http://www.sanger.ac.uk/cosmic/) was used to determine the frequency of somatic mutations in *RINT1* in colorectal cancers. This database included The Cancer Genome Atlas dataset as well as other available datasets. Once the colorectal cancer cases containing somatic mutations in *RINT1* were identified, an analysis of the nature of these mutations (e.g. nonsense, missense, etc.) was performed.

### Generation of *Rint1* variant expression constructs

An expression vector containing a FLAG-tagged full-length human *RINT1* cDNA was generously provided by Dr. Mitsuo Tagaya (Tokyo University of Pharmacy and Life Sciences, Tokyo, Japan; Arasaki et al. 2006). Missense variant alleles in *RINT1* were generated using the QuickChange II XL kit (Agilent Technologies) according to the provided protocol. Three missense variant alleles were generated: *RINT1* n.456A>G (I152M), *RINT1* n.503T>C (I168T), and *RINT1* n.456A>G; n.503T>C (I152M and I168T). These missense alleles are identical to the variants found in the B6 mouse strain and are found in the coiled coil domain and are thus similar to many of those associated with Lynch syndrome type cancers.

Briefly, primers were designed to change nucleotide 456 from an adenine to a guanine (456g forward primer: 5’-GAACCATGATTAGCCAGATGGAAGAGATCGAACGTCATC-3’; 456g reverse primer: 5’-GATGACGTTCGATCTCTTCCATCTGGCTAATCATGGTTC-3’) and nucleotide 503 from a thymine to a cytosine (503c forward primer: 5’-CATCTTGCTTACCTTAAATGGATTTCACAAACTGAAGAACTAAGTGAT-3’; 503c reverse primer: 5’-ATCACTTAGTTCTTCAGTTTGTGAAATCCATTTAAGGTAAGCAAGATG-3’). Plasmids containing the individual variants were generated via PCR amplification and used to transform XL10-Gold ultra-competent *E*. *coli* cells, which were then plated on LB agar plates containing 10 μg/ml Ampicillin (Fisher Scientific, Pittsburg, PA). Single colonies were picked for overnight growth in liquid media and plasmids isolated using Qiagen Mini- or Maxi-prep kits (Qiagen, Valencia, CA). The plasmid containing both variants was generated by PCR using one of the single variant plasmids and the other set of site specific mutation-inducing primers. The resulting plasmids were transformed into XL10-Gold ultra-competent *E*. *coli* cells and isolated as described above. The sequence of all *RINT1* construct inserts were confirmed via Sanger Sequencing at the UNMC DNA Sequencing Core Facility.

### Cell culture and transfection

The pCl-neo-HA-hUVRAG plasmid was a gift from Dr. Noboru Mizushima (Addgene plasmid #24290; Itakura et al. 2008). Each RINT1 expression plasmid, with and without the UVRAG plasmid, was transfected into HEK293T cells using the Lipofectamine 2000 reagent (Life Technologies, Grand Island, NY) according to the manufacturer’s instructions. Cells were grown for 24 hours after transfection. Cell lysates were made using TNE lysate buffer (10 mM Tris acetate, 0.5% NP-40, 2 mM EDTA) containing 10 μg/ml Phenylmethanesulfonyl fluoride (PMSF; Sigma-Aldrich, St. Louis, MO) and Protease inhibitor cocktail (Sigma-Aldrich). Lysates were quantified using the Pierce BCA Protein Assay Kit (Pierce Biotechnology, Rockford, IL).

### Immunoprecipitation and western blotting

Equal amounts of whole cell lysate from cell cultures transfected with the various *RINT1* constructs were used for immunoprecipitation. Anti-FLAG M2 affinity gel (Sigma-Aldrich) was used to pull down the FLAG-tagged RINT1 protein, along with any bound proteins, according to the manufacturer’s instructions. Briefly, agarose beads were incubated with whole cell lysate overnight, washed to remove non-bound proteins, and then incubated with 15 μg of FLAG peptide (F3290, Sigma-Aldrich) to elute FLAG-tagged RINT1. Supernatant containing the eluted RINT1 protein, along with any proteins bound to RINT1, was removed from beads and mixed with Laemmli sample buffer (Bio-Rad, Hercules, CA).

Immunoprecipitated samples and whole cell lysates were loaded onto polyacrylamide gels. After electrophoresis, proteins were transferred to nitrocellulose membranes (GE Healthcare Bio-Sciences, Pittsburgh, PA) and blocked in 50% Odyssey Blocking buffer (LI-COR Biosciences, Lincoln, NE) diluted in PBS. Membranes were incubated overnight at 4°C with the following antibodies RINT1 (1:400 dilution, #19406, Santa Cruz Biotechnologies, Dallas, TX), ZW10 (1:2500 dilution, ab21582, Abcam, Cambridge, MA) or UVRAG (3:5000, Sigma-Aldrich, St. Louis, MO). Membranes were washed four times in PBS containing 0.1% Tween 20, then incubated with secondary antibodies (Alexa Fluor 680 donkey anti-goat IgG (1:2500 dilution, A21084, Life Technologies) and Odyssey donkey anti-rabbit IRDye 800CW (1:2500 dilution, 926–32213, LI-COR Biosciences)). Membranes were imaged using the Li-COR Odyssey Infrared Imaging System (Li-COR Biosciences) and quantification was performed using Image Studio software, version 4.0 (Li-COR Biosciences). Statistical significance for relative abundance of immunoprecipitated materials was determined using the independent samples t-test in SPSS.

## Results

### Genetic background alters spontaneous tumorigenesis in *Rint1*^*+/-*^ mice

The original purpose of this study was to explicitly evaluate the impact of heterozygosity for the *Rint1* knockout allele on tumorigenesis in *Apc*^*Min*/+^ mice. The *Rint1*^*+/-*^ mice we obtained were on a mixed 129 x B6 genetic background. Because 129 strains carried alleles that modify the intestinal tumor phenotype in *Apc*^*Min/+*^ mice, we needed to place the *Rint1* knockout allele onto a pure B6 background before we could cross *Rint1*^*+/-*^ mice with *Apc*^*Min/+*^ mice.

We observed that the risk of spontaneous tumor development in *Rint1*^*+/-*^ mice declined progressively during the process of backcrossing to the B6 strain. Consistent with the previous report indicating that ~80% of *Rint1*^*+/-*^ mice developed tumors, we found that three of the four (75%) *Rint1*^*+/-*^ founder mice on the mixed 129 x B6 genetic background developed spontaneous tumors and required euthanasia by ~400 days of age (Table 2). The spectrum of tumor types observed in these mice was consistent with that reported previously in *Rint1*^*+/-*^ mice (Lin et al. 2007). The fourth *Rint1*^*+/-*^ mouse died unexpectedly and could not be evaluated for tumor development. By contrast, tumors were observed in just 28.5% (2 of 7) of the *Rint1*^*+/-*^ mice from the F1 (N1) generation, which was produced by crossing the original founders with B6 mice. No tumors were found in a group of age matched, *Rint1*^*+/+*^ F1 (N1) mice. In the next backcross generation to the B6 strain (N2), none of the *Rint1*^*+/-*^ mice (0 of 13) or *Rint1*^*+/+*^ mice (0 of 9) developed tumors by 450 days of age. Furthermore, no tumors were observed in *Rint1*^*+/-*^ mice analyzed from any generation thereafter. At the N2 generation, the *Rint1*^*+/-*^ mice carried a 129 allele at, on average, 6% of markers unlinked to the *Rint1* knockout; this residual heterozygosity represents approximately quarter of that present in the founder mice. A plausible interpretation of these data is that spontaneous tumorigenesis in *Rint1*^*+/-*^ mice is strongly influenced by genetic background.

**Table 2:** Impact of Genetic Background on Spontaneous Tumorigenesis in *Rint1*^*+/-*^ mice.

B6.129Sv-*Rint1*^*tm1WHL*^ Generation	Genotype	Percentage of mice developing tumors	Location of tumors (# tumors/#tumor bearing mice)
B6.129Sv-*Rint1*^*tm1WHL*^ Founders	*Rint1*^*+/-*^	75% (3/4)	Endometrium (1/3)
			Lung (2/3)
			Liver (1/3)
			Thymus (1/3)
(B6 x B6.129Sv-*Rint1*^*tm1WHL*^) F1	*Rint1*^*+/-*^	17% (2/12)	Lung (2/2)
			Liver (1/2)
	*Rint1*^*+/+*^	0% (0/5)	N/A^1^
(B6 x B6.129Sv-*Rint1*^*tm1WHL*^) N2	*Rint1*^*+/-*^	0% (0/16)	N/A
			N/A
	*Rint1*^*+/+*^	0% (0/9)	N/A

^1^ N/A = Not applicable.

Homozygosity for the *Rint1* knockout was reported to result in embryonic lethality on a mixed 129 x B6 background (Lin et al. 2007). To determine if genetic background also impacted this phenotype, we intercrossed a small number of *Rint1*^*+/-*^ mice. From these intercrosses, only *Rint1*^*+/+*^ and *Rint1*^*+/-*^ mice were recovered (Table 3). The failure to recover *Rint1*^*-/-*^ mice from this cross indicates that even on the B6 background, homozygosity for the *Rint1* knockout allele leads to embryonic lethality.

**Table 3:** Intercrossing B6.*Rint1*^*+/-*^ mice yields no *Rint1*^*-/-*^ progeny.

**Genotype**	**Number Expected**	**Number Observed**^**a**^
*Rint1*^*+/+*^	~16	25
*Rint1*^*+/-*^	~32	38
*Rint1*^*-/-*^	~16	0^b^

^a^ 63 progeny were obtained from *Rint1*^+/-^ intercrosses.

^b^ p = 1.3 x10^-5.^

### Heterozygosity for the *Rint1* knockout allele does not impact tumor development in B6.*Apc*^Min/+^ mice

**Fig 2:**
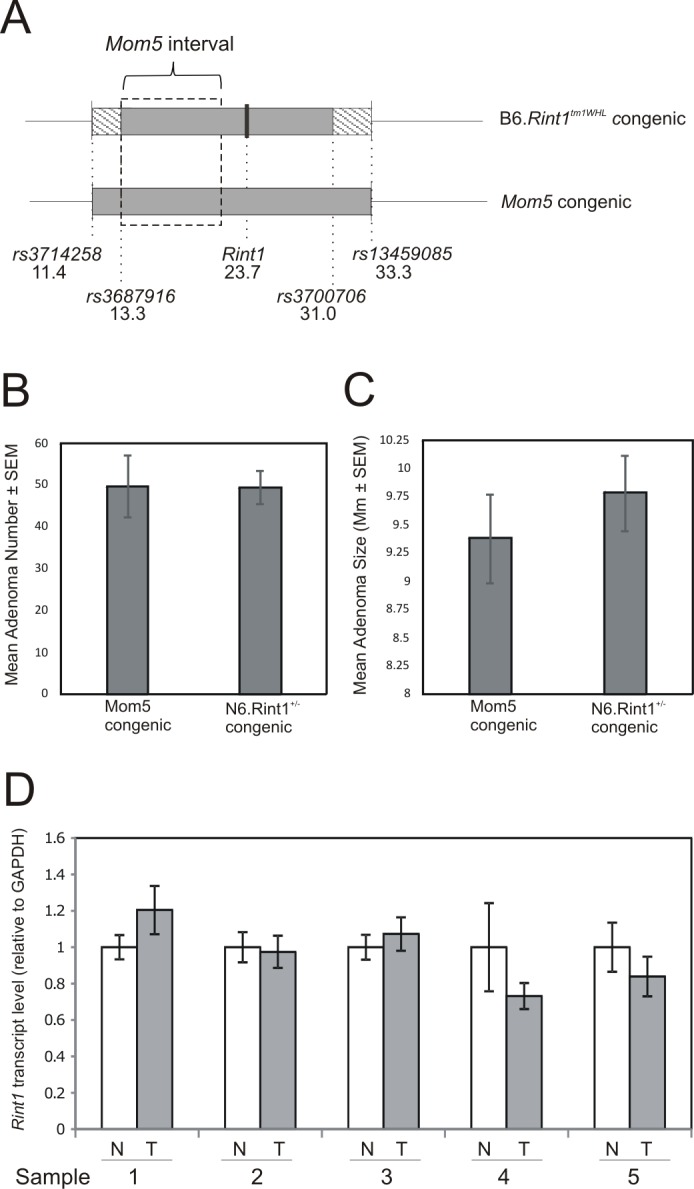
Loss of *Rint1* and tumorigenesis in B6. *Apc^Min/+^* mice. After marker assisted selection, the B6.*Rint1*^*+/-*^ strain was uniformly B6 except for a 21.9 Mbp interval of residual heterozygosity spanning the *Rint1* knockout allele (A). This region includes the entire *Mom5* modifier interval (A). Mean intestinal tumor number (B) in *Rint1*^*+/+*^; *Apc*^*Min*/+^ mice (N = 25) and *Rint1*^*+/-*^; *Apc*^*Min*/+^ mice (N = 12) is shown. Mean diameter of intestinal tumors (C) in *Rint1*^*+/+*^; *Apc*^*Min*/+^ and *Rint1*^*+/-*^; *Apc*^*Min*/+^ mice (N = 33–62 tumors per mouse from N = 7 representative mice of each genotype) is shown. Analysis of *Rint1* expression in normal colon and colon tumors (N = 5 pairs) in *Apc*^*Min*/+^ mice revealed no evidence of loss of *Rint1* (D).

After five generations (N5) of backcrossing to the B6 strain in conjunction with marker assisted selection, all the segregating 129 alleles not on chromosome 5 had been eliminated from the B6.129-*Rint1*^tm1WHL^ strain (hereafter referred to as B6.*Rint1* strain). Further genome wide analysis confirmed that the only region of residual heterozygosity was on chromosome 5 and comprised a 21.9 Mbp interval spanning the *Rint1* knockout allele (at 23.7 Mbp). This region of heterozygosity was located between 11.4 and 33.3 Mbp (Fig 2A) and contained the *Mom5* locus, which has been localized to the 8 Mbp interval from 13.25 to 21.35 Mbp (Fig 2A) [14]. Thus, *Rint1*^*+/-*^ mice were also heterozygous for the *Mom5* locus (*Mom5*^*129/B6*^). Because the *Rint1* knockout allele is tightly linked to the 129 allele of *Mom5*, which is associated with reduced tumor multiplicity in *Apc*^*Min/+*^ mice, to assess the impact of heterozygosity for the *Rint1* knockout on tumorigenesis in *Apc*^*Min/+*^ mice, we needed to compare *Rint1*^*+/-*^;*Mom5*^*129/B6*^;*Apc*^*Min*/+^mice with *Rint1*^*+/+*^;*Mom5*^*129/B6*^*;Apc*^*Min*/+^ mice. Therefore, *Rint1*^*+/-*^ mice, heterozygous for the 21.9 Mbp interval spanning both the *Mom5* modifier locus and the *Rint1* knockout allele, were compared to mice from the *Mom5* congenic strain, which is heterozygous for 129 alleles through a 46.4 Mbp interval that completely encompasses the 21.9 Mbp interval carried in the B6.Rint1 strain [14].

Our analysis indicated that heterozygosity for *Rint1* had no discernible impact on tumorigenesis in *Apc*^*Min*/+^ mice. Mean tumor multiplicity in the *Rint1*^+/-^; *Mom5*^*129/B6*^;*Apc*^*Min*/+^ mice was 49.6±4, which was essentially identical to the mean of 49.8±7.4 in *Rint1*^+/+^; *Mom5*^*129/B6*^*;Apc*^*Min*/+^ mice (Fig 2B; p = 0.47). These data indicate that heterozygosity for the *Rint1* knockout allele did not impact tumor number in *Apc*^*Min*/+^ mice. Likewise, heterozygosity for the *Rint1* knockout allele did not impact mean tumor diameter in *Apc*^*Min*/+^ mice (Fig 2C). Since *Rint1*^*-/-*^ mice do not survive, we could not examine the impact of homozygosity for the *Rint1* knockout allele on tumorigenesis in *Apc*^*Min*/+^ mice. Nevertheless, we explored the possibility that the remaining wild type allele of *Rint1* might be lost somatically in tumors that develop in *Rint1*^+/-^; *Apc*^*Min*/+^ mice. Quantitative PCR analysis of a small number of colon tumors from *Rint1*^+/-^; *Apc*^*Min*/+^ mice indicated that the wild type allele of *Rint1* was not lost somatically in these tumors (data not shown) and *Rint1* expression is not reduced in colon tumors in *Apc*^*Min*/+^ mice (Fig 2D). Although a small fraction of *Apc*^*Min*/+^ mice spontaneously develop mammary tumors (Moser et al. 1993), this phenotype was not enhanced in *Rint1*^+/-^; *Apc*^*Min*/+^ mice (data not shown).

### *RINT1* overexpression and somatic missense mutations are associated with colorectal cancer

**Fig 3:**
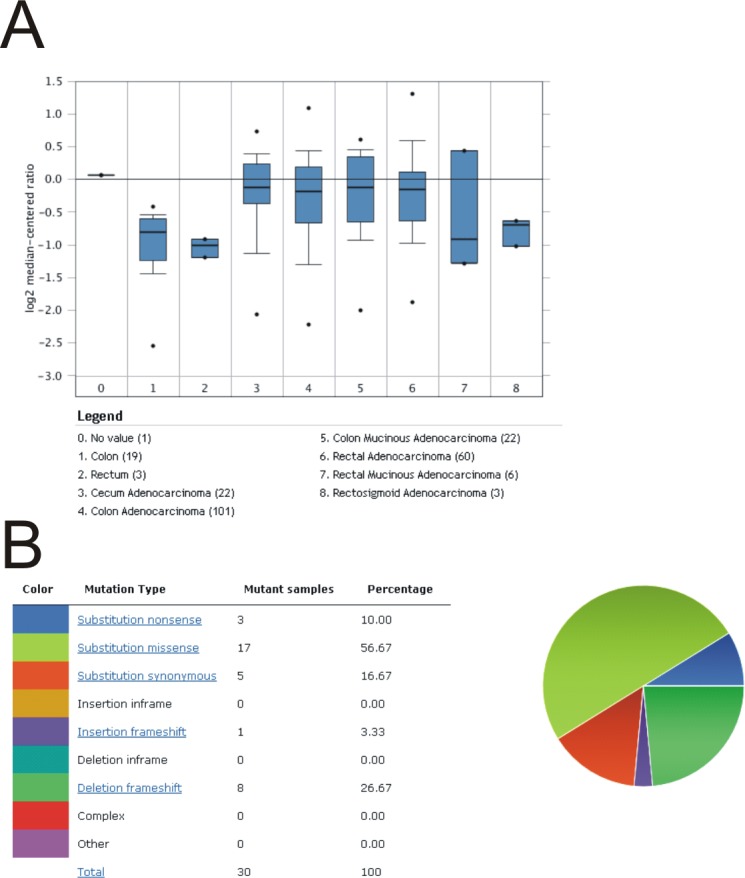
Bioinformatic analysis of *RINT1* expression and mutation spectra in human colorectal cancers. Information regarding relative expression of *RINT1* in normal colorectal tissue and colorectal cancers was extracted from the ONCOMINE database (A). Analysis of the nature of somatic mutations in *RINT1* found in colorectal cancers was extracted from the COSMIC database (B).

Our studies with the *Rint1* knockout did not support the hypothesis that heterozygosity for the *Rint1* knockout allele promotes tumorigenesis in *Apc*^*Min*/+^ mice. These observations were consistent with data extracted from the COSMIC database, which revealed no evidence for loss of heterozygosity or deletion of *RINT1* in colorectal cancers, suggesting that somatic loss of *RINT1* was rare during colorectal tumorigenesis. Furthermore, we note that just 0.16% (1 of 607) colorectal tumors in the COSMIC database showed loss of *RINT1* expression. By contrast, 6.1% (37 of 607) of colorectal tumors in the COSMIC database overexpressed *RINT1*. Likewise, comparison of colorectal cancers and normal mucosa in the Oncomine database showed that *RINT1* transcript levels were significantly greater in many colon adenocarcinoma subtypes (Fig 3A). Somatic mutations in *RINT1*were detected in 2% (30 of 1493) of cancers of the large intestine, and ~57% (17/30) of these somatic mutations were missense mutations (Fig 3B), a phenomenon that is more characteristic of an oncogene than a tumor suppressor gene. Altogether, the expression and somatic mutation spectrum data were more consistent with the idea that RINT1 might function as an oncogene in the context of colon carcinogenesis rather than a tumor suppressor gene. As mentioned previously, germline variants in the human *RINT1* gene have been associated with increased risk of various cancers associated with Lynch Syndrome, including intestinal cancers[10]. The majority of these cancer-associated germline variants in RINT1 are missense variants.

### Missense variants in *RINT1* impact protein-protein interactions *in vitro*

**Fig 4:**
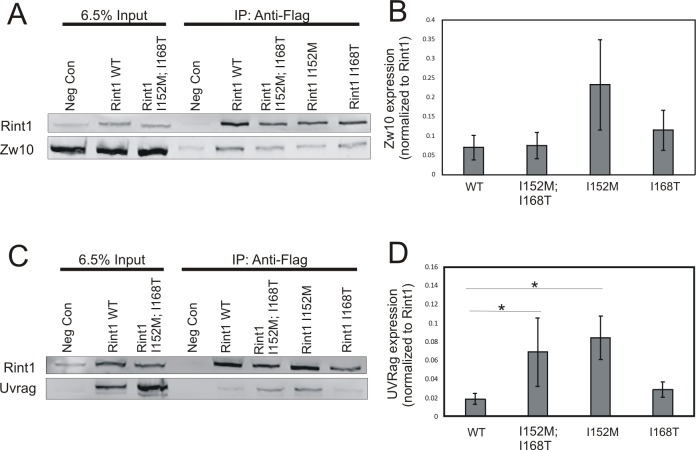
*In vitro* characterization of missense variants in *RINT1*. FLAG-tagged *RINT1* mutant plasmids were transfected into HEK293T cells and an anti-FLAG antibody was then used to immunoprecipitate RINT1 and interacting proteins from cell lysate. Cells transfected with empty vector served as the negative control (Neg Con). Cells transfected with the RINT1 wildtype (WT) expression vector served as the positive control. A representative immunoblot for ZW10 immunoprecipitation is shown (A). Quantification of the ZW10 immunoprecipitation blots (N = 5) are shown (B). A representative immunoblot for UVRAG immunoprecipitation is shown (C). Quantification of the UVRAG immunoprecipitation blots (N = 4) are shown (B). The asterisk indicates differences with a p ≤ 0.05.

If the *RINT1* missense variants function as oncogenes, we would anticipate that these variant alleles would act in at least a partially dominant manner to promote tumorigenesis. Given the fact that a number of *RINT1* missense variants are found within the coiled-coil protein interaction domain, we postulated that *RINT1* missense variants might alter the interaction of RINT1 with some of its binding partners. The coiled-coil domain of RINT1 mediates the interaction of RINT1 with a number of proteins involved in vesicle trafficking between the ER and Golgi, including ZW10 and UVRAG. To evaluate the potential impact of *RINT1* missense variants on protein-protein interactions, we generated *RINT1* expression vectors from which a FLAG-tagged wild type allele of *RINT1* as well as the variant *RINT1* I152M allele, *RINT1* I168T allele, or the *RINT1* I152M; I168T allele could be expressed transiently. Each *RINT1* plasmid was transfected into HEK293T cells and RINT1-containing complexes from cell lysates were immunoprecipitated with an anti-FLAG antibody. The ability of each FLAG-tagged RINT1 protein to co-immunoprecipitate the RINT1-interacting proteins, ZW10 and UVRAG was assessed. The ability of the RINT1 variant proteins to co-immunoprecipitate endogenous ZW10 did not differ from that of the wild-type RINT1 protein (Fig 4A). Evaluation of these same *RINT1* variants in a UVRAG co-immunoprecipitation assay was also performed. For these experiments a *UVRAG* plasmid was co-transfected with the *RINT1* plasmids due to the inability of the antibody to detect the endogenous UVRAG in these cells. In this series of studies, we consistently observed a ≥ 2-fold increase in UVRAG co-immunoprecipitation for the I152T and I152T;I168T RINT1 variant proteins compared to the RINT1 wild-type protein (Fig 4B). These observations suggest that missense alleles of *RINT1* can display altered protein-protein interactions.

## Discussion

It has been reported previously that mice heterozygous for the *Rint1* knockout allele spontaneously develop lymphomas as well as solid tumors. These studies were performed on a mixed 129 x B6 genetic background, and we too found that on this mixed genetic background, *Rint1*^*+/-*^ mice developed a variety of solid tumors. However, this tumor susceptibility was lost as we backcrossed the *Rint1* knockout onto the B6 background. These data suggest that the genetic background may influence the penetrance of the tumor phenotype associated with heterozygosity for the *Rint1* knockout allele. However, an alternative explanation of these data is also plausible. We note that in the original description of the tumor susceptibility in *Rint1*^*+/-*^ mice, the majority of the mice (29%) developed lymphomas [2]. Since these *Rint1*^*+/-*^ mice were on a highly mixed 129 x B6 genetic background, which is known to have a high incidence of spontaneous lymphoma development[16], we cannot exclude the possibility that at least some of the tumor predisposition in *Rint1*^*+/-*^ mice observed in Lin et al is attributable to genetic background rather than the *Rint1* knockout allele. This possibility cannot be excluded because this original description of the tumor phenotype in *Rint1*^*+/-*^ mice did not include a parallel analysis of tumorigenesis in *Rint1*^*+/+*^ mice on this same mixed genetic background. Because of the possibility that genetic background might impact the tumor phenotype in *Rint1*^*+/-*^ mice, we sought to determine if genetic background might also impact the embryonic lethality associated with homozygosity for the *Rint1* knockout allele. However, as was observed on the mixed 129 x B6 genetic background, homozygosity for the *Rint1* knockout allele on a pure B6 genetic background also resulted in embryonic lethality.

The original, primary purpose of this study was to evaluate the impact of heterozygosity for a knockout of *Rint1* on tumorigenesis in *Apc*^*Min*/+^ mice. However, this analysis revealed that heterozygosity for *Rint1* did not impact intestinal tumor number or size. We also found no evidence for somatic loss of *Rint1* in colon tumors in *Apc*^*Min*/+^ mice. Altogether, our studies with *Rint1*^*+/-*^ mice did not support the idea that the *Rint1* knockout allele functioned as either a modifier allele of tumorigenesis in *Apc*^*Min/+*^ mice or a cancer predisposing allele on the B6 background. Thus, these studies did not provide robust evidence to support the hypothesis that *Rint1* functions as a tumor suppressor. Likewise, analysis of data available in the COSMIC and ONCOMINE databases failed to yield strong evidence that loss of *RINT1* was associated with intestinal carcinogenesis in humans. Rather, analyses of available colorectal cancer data in these databases suggested that overexpression of *RINT1* as well as somatic missense mutations in *RINT1* may be associated with colorectal carcinogenesis. It has been reported previously that germline missense variants in *RINT1* are associated with increased risk of breast and Lynch Syndrome type cancers [10]. However, the impact of these various somatic and germline *RINT1* mutations, most of which are missense mutations, on the function of RINT1 is not known.

To explore the possible impact of RINT1 missense variants on RINT1 function, we expressed several missense *RINT1* variants *in vitro* and examined the impact of these variants on the interaction of RINT1 with some of its binding partners. Compared to wild type, some of the *RINT1* variants we generated showed a more robust association with UVRAG, a known RINT1 interacting protein that is involved in retrograde transport and autophagy [17]. The RINT1 variants we examined *in vitro* are similar to some of those associated with Lynch Syndrome type cancers in that they are missense variants in the coil-coil domain, which is thought to mediate the interaction of RINT1 with many of its binding partners. Thus, our results raise the possibility that missense mutations in *RINT1* may represent dominant, gain of function alleles that may be functionally equivalent, at least in a certain respect, to *RINT1* overexpression. Altogether, these data are more consistent with the hypothesis that *RINT1* functions as an oncogene rather than a tumor suppressor gene in the context of colorectal cancer.
